# Physiotherapy in Text Neck Syndrome: A Scoping Review of Current Evidence and Future Directions

**DOI:** 10.3390/jcm14041386

**Published:** 2025-02-19

**Authors:** Joanna Piruta, Wojciech Kułak

**Affiliations:** 1 Doctoral School, Medical University of Bialystok, 15-089 Bialystok, Poland; 2 Department of Pediatric Neurology and Rehabilitation, Medical University of Bialystok, 15-274 Bialystok, Poland

**Keywords:** text neck syndrome, physiotherapy, rehabilitation, smartphone overuse, musculoskeletal disorders, neck pain, mobile phone users

## Abstract

**Background:** Musculoskeletal disorders associated with excessive smartphone use represent a significant health issue. Text neck syndrome is one such disorder within that group, increasingly affecting individuals worldwide across various age groups. The phenomenon of text neck may occur in individuals who frequently and for prolonged periods adopt a forward-flexed neck and head position while looking at the screens of mobile electronic devices. Various therapeutic methods are used in the treatment of text neck syndrome. However, there is no consensus on text neck rehabilitation, which poses a challenge for physiotherapists. **Objective:** The aim of this study is to analyze the phenomenon of text neck, with a particular emphasis on current scientific reports regarding the rehabilitation of text neck syndrome. The scoping review was conducted to determine the physiotherapy methods currently used in the treatment of individuals with text neck, assess their impact on symptom reduction, and identify existing knowledge gaps and limitations in the current literature on the rehabilitation of text neck syndrome. **Design:** A scoping review was conducted on the treatment of text neck syndrome based on electronic databases: PubMed, ResearchGate, Physiotherapy Evidence Database (PEDro), and the Cochrane Library. The databases were searched up to 1 December 2024. The inclusion criteria comprised studies investigating physiotherapy interventions for individuals with text neck, published between 2018 and 2024 and written in English. **Results:** A total of fifteen papers were reviewed, focusing on various methods used in text neck rehabilitation, including postural correction exercises, stabilization exercises, strengthening and stretching exercises, Pilates, PNF (Proprioceptive Neuromuscular Facilitation), kinesiology taping, Bowen therapy, and manual therapy. Nearly all studies were conducted in the adult population (93%), with the majority of studies taking place in India (60%). **Conclusions:** In summary, all studies suggest that appropriate physiotherapeutic interventions can provide significant benefits, including pain reduction, posture correction, and improved range of motion in the cervical spine. The best outcomes appear to be achieved by combining various therapeutic techniques. However, further high-quality research is needed to strengthen the evidence and offer reliable recommendations for clinical practice. Additionally, there is limited research on physiotherapy for text neck in the pediatric population, presenting a potential area for future studies.

## 1. Introduction

The advancement of technology has made nearly everyone an owner of a device such as a mobile phone [1]. In 2022, approximately 83% of people worldwide were estimated to own a mobile phone [2]. In 2024, 7.21 billion people are indicated to be smartphone users, accounting for 90% of the global population [3]. In the United States, as many as 97% of residents aged 18–29 are smartphone owners [3]. According to statistics, approximately 79% of people aged 18–44 virtually never part with their mobile phones, and 3.5 billion smartphone owners exhibit signs of addiction to those devices [4,5,6].

Undoubtedly, smartphones offer many attractive features. Mobile phones are no longer used solely for communication. They have become devices that provide entertainment, enable photography, allow users to stay updated with the latest news, and serve as tools for work and learning [7,8,9]. Unfortunately, despite their many advantages, prolonged screen time on smartphones may lead to numerous adverse health consequences [10]. Excessive use of smartphones may negatively affect sleep quality, cause vision problems (e.g., eye strain, blurred vision), lead to symptoms related to the peripheral nervous system, contribute to psychosocial issues (e.g., addiction, social isolation, depression), and also adversely impact posture [11,12,13,14,15,16].

Almost every day, one can observe people who, while using electronic devices, thrust their heads forward or maintain their cervical spine in a flexed position, staring at a smartphone screen for many hours throughout the day. Unfortunately, such behavior may adversely impact human health, leading to adverse consequences, such as musculoskeletal strain and the onset of neck and cervical pain [17,18]. Neck pain radiating to the upper limbs and headaches constitute a significant problem that affects the quality of life and limits daily functioning [19]. Neck pain affects many people worldwide across various age groups [20]. That issue also affects increasingly younger individuals, partly due to the more frequent and prolonged use of smartphones [21]. The prevalence of spinal pain among young adults is on the rise [22]. That phenomenon is influenced by many factors, one of which is the excessive use of computers and smartphones during leisure time [22]. It is estimated that the prevalence of neck pain in groups aged 7–11 years who are spending around 5–8 h daily using electronic devices is around 70% [23].

According to statistical data, nearly half of the people in the USA in 2024 spent about 5–6 h daily using a smartphone, which translates to 1825–2190 h annually (2.5–3 months per year spent solely on smartphone usage) [3]. A reduction in the time dedicated to physical activity, a sedentary lifestyle, lack of movement, and prolonged maintenance of static positions contribute to the occurrence of musculoskeletal disorders and spinal pain [19,20]. It is estimated that the prevalence of musculoskeletal disorders among smartphone users ranges from 50% to 84%, particularly affecting areas such as the neck, shoulders, upper back, and upper body [19]. One such disorder is the so-called ‘text neck syndrome’ [24]. The prevalence of text neck syndrome ranges from 16.7% to 93.2% [20,23,25,26,27,28,29,30,31,32,33].

The findings highlight the urgent need to address the issue of text neck syndrome. Various therapeutic methods are used in the treatment of text neck syndrome. However, there is no consensus on text neck rehabilitation, which poses a challenge for physiotherapists. The aim of this study is to analyze the phenomenon of text neck, with a particular emphasis on current scientific reports regarding the rehabilitation of text neck syndrome. The scoping review was conducted to determine the physiotherapy methods currently used in the treatment of individuals with text neck, assess their impact on symptom reduction, and identify existing knowledge gaps and limitations in the current literature on the rehabilitation of text neck syndrome.

Conducting a scoping review allows for the collection and summary of available studies related to the rehabilitation of text neck syndrome, providing a better understanding of the current state of knowledge regarding physiotherapeutic methods for treating this condition. This review helps identify areas where research is lacking or requires further exploration. It can guide future studies towards the most important aspects of text neck syndrome treatment that have not yet been sufficiently examined.

## 2. Text Neck Syndrome

### 2.1. Definition

Staring at the screen of a mobile device, such as a smartphone, for many hours a day with a flexed head and neck position predisposes to the development of text neck syndrome [24]. The term ‘text neck’ was first used by chiropractor Dr. L. Fischman [24]. Dr. Fischman noted that the tension associated with prolonged, repetitive, and frequent maintenance of a flexed neck and head position while using electronic devices such as smartphones or tablets leads to strain, injuries, and stiffness in the cervical spine area [8,34,35]. Text neck syndrome may be referred to as: ‘turtle neck posture’, ‘smartphone neck syndrome’, ‘tech neck’, ‘SMS neck’, or ‘iNeck’ [8,36]. The text neck posture is characterized by an increased flexion of the cervical spine and is caused by activities that require looking down, below eye level, such as when typing text messages [12]. Depending on the degree of progression, the text neck syndrome may present with a varied clinical picture [37].

The diagnosis of text neck syndrome is based on subjective (interview) and objective examinations, which include, among others, assessment of posture, pain intensity (e.g., using the Numerical Pain Rating Scale—NPRS or Visual Analogue Scale—VAS), disability (e.g., using the Neck Disability Index), muscle tension, and joint range of motion [5,38]. In some cases, imaging studies are recommended to rule out the presence of other medical conditions [38]. When diagnosing text neck syndrome, it is important to exclude the following conditions and diseases as the cause of the symptoms: osteoporosis, stenosis, scoliosis, spondylolisthesis, torticollis, spinal infections or inflammatory disorders, malignancies, herniation, and trauma [19,39].

In order to diagnose text neck syndrome, the following criteria are taken into account:

- Using the phone for 3 h or more per day;
- The presence of 3 out of the following 6 symptoms: neck pain, upper back pain, shoulder pain, headache, insomnia, tingling and numbness in the upper limbs;
- Using the phone with a neck flexion of approximately 15 degrees or greater [6].

These diagnostic criteria are taken into account when diagnosing text neck syndrome. However, it should be noted that clear diagnostic criteria for text neck syndrome have not yet been established.

### 2.2. Symptomatology

Correct posture ensures proper balance and functioning of the body. However, when there is a disturbance in one segment, it may lead to abnormalities and disorders in other parts of the body. Frequent use of smartphones causes the head and neck to often tilt forward toward the screen of the mobile device. That frequent adoption of an incorrect and burdening position may contribute to an increased risk of musculoskeletal problems in the neck and upper torso [19,40]. The set of symptoms associated with smartphone use is referred to as text neck syndrome [41]. In that musculoskeletal disorder, a muscular imbalance occurs. A correlation has been observed between smartphone addiction and a decrease in muscle strength and endurance [19]. Particularly, the deep neck flexor muscles become weakened [19]. Such a static flexed position leads to the overloading of the suboccipital muscles, the erector spinae, the levator scapulae, the trapezius, and the semispinalis muscles [37]. Tension in the suboccipital muscles may result in the onset of tension-type headaches [37].

In individuals with text neck syndrome, changes in posture and structural overload may occur. However, these are usually functional changes rather than structural ones in the sense of permanent, irreversible deformities. Prolonged strain on the neck can lead to excessive muscle tension, changes in the natural curvature of the cervical spine (e.g., flattening of cervical lordosis), pain and discomfort, and, in the long run, possible damage to spinal structures [5,23,42]. With prolonged flexion, continuous stress may result in the loosening of the posterior ligaments, instability of the vertebral segments, and degenerative joint disease, potentially causing plastic changes within the nervous system and resulting in clinical symptoms [12]. Neurological symptoms are additional manifestations that can occur in text neck syndrome. These may include tingling, numbness, or weakness in the arms or hands, often due to nerve compression or irritation in the cervical spine. While these symptoms are less common, they may indicate that the condition has progressed to affect the nerves [12,43].

The reinforcement of poor movement habits and the lack of correction of incorrect posture over time may lead to limited mobility in the cervical spine and further segments, disturbances in spinal curvature, reduced lung capacity, as well as the onset of neck pain, radiating pain to the upper limbs, and even numbness in the hands [24,34,35,44,45]. An unnatural, forced position with a tilted head puts excessive strain on the spine structures, eventually leading to the development of a herniated disc and degenerative changes [19,46]. Untreated text neck syndrome may lead to inflammation in the ligaments, muscles, or nerves [8]. The consequence in the future may be permanent changes in the musculoskeletal system [7]. Table 1 presents the symptoms and consequences of text neck syndrome.

### 2.3. Pathophysiology

**Figure 1:**
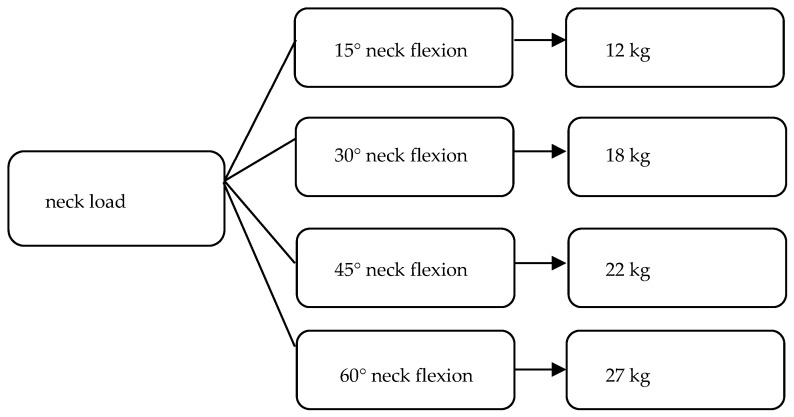
Distribution of the load exerted on the cervical spine depending on the angle of neck flexion. Own elaboration based on [4,11,40].

Thanks to the specific structural formation of the spine, it is possible to perform the appropriate functions [7]. The intervertebral disc is thicker at the front than at the back [2,5]. That structure of the disc, combined with the shape of the cervical vertebral bodies, creates cervical lordosis [46]. Thanks to the intervertebral discs and cervical lordosis, a cushioning function and a greater range of backward flexion are possible [35]. In an adult person in a neutral anatomical position, the cervical spine supports the weight of the head, which is approximately 5 kg [11]. The greater the flexion in the cervical spine, the greater the load placed on the spine [11]. Figure 1 shows the distribution of the load exerted on the cervical spine depending on the angle of neck flexion.

Prolonged, repetitive maintenance of a flexed position while using a mobile phone leads to the forward shift of the head’s center of gravity [7,8]. Incorrect head positioning and prolonged positions with the head jutting forward, such as when using a mobile phone, increase the load on the spine joints and affect the length and endurance of the muscles [9]. Frequent, repetitive positioning of the neck in a flexed position with forward head protrusion may excessively stimulate the muscles, leading to muscular imbalance, functional changes, and overload [51]. Increased tension in specific muscles and a greater angle of neck flexion may lead to proprioceptive disturbances, limited mobility in the cervical spine, and referred pain to certain parts of the body, which may be caused, among other factors, by trigger points within the affected muscles [1]. In text neck syndrome, increased strain and stiffness are observed in the upper part of the trapezius muscle, the neck extensor muscles, and weakness of the deep neck flexor muscles [9,12,52]. Muscle imbalance and incorrect curvature of the cervical spine may lead to microdamage of soft tissues, overload of spinal structures, and the onset of pain [53,54].

### 2.4. Risk Factors

Risk factors that predispose to the onset and intensification of symptoms of text neck syndrome include the following:

- Frequency and duration of time spent in an improper position while using the phone;
- Position while using the smartphone;
- Height at which the mobile device is held;
- Lack of physical activity;
- Smartphone addiction;
- Female gender [12,39,55,56].

As reported by Elvan A. et al. [57], using a mobile phone for more than 3 h a day is a significant risk factor for the development of text neck syndrome. Studies conducted by Paek K.S. [58], Gong W. et al. [59], and Choi J.H. et al. [60] have indicated that a prolonged flexed position in the cervical spine negatively affects the endurance of the deep neck flexor muscles. Ayhualem S. et al. [1], in their publication, draw attention to the increased risk of neck pain in individuals who spend much time using smartphones. Shahzad Y. et al. [46] and Khan S. et al. [2] have demonstrated that neck pain occurs more frequently in individuals who spend more than 4 h a day using a mobile phone. De Vitta A. et al. [61], in a study conducted with a group of 1143 students, have also observed a correlation between the duration of smartphone use and the frequency of neck pain.

A study conducted by Sarraf F. and Varmazyar S. [18] confirms that the position adopted while looking at the screen of a mobile device affects the activity and degree of muscle strain. Researchers have shown that the best head and neck position is achieved when sitting with back support, while the worst position is sitting without back support [18]. The posture has an impact on the amount of strain placed on the cervical spine [18]. The lack of arm support while using a phone negatively affects the alignment of the cervical spine [62,63].

A study conducted by Raihan H.A. and Rahman F. [52] has demonstrated the relationship between the occurrence of text neck syndrome and the height at which the phone is held during use. According to Kokiwar P.R. et al. [34], neck pain symptoms are most commonly reported by individuals who hold their smartphones at the level of their abdomen. A similar correlation has been observed in studies conducted by Rashid M.K. et al. [26], where neck pain had been more commonly observed in individuals holding their smartphones with their necks bent at approximately 60 degrees. In turn, Correia I.M.T. et al. [64] have not confirmed any correlation between the body posture associated with text neck syndrome and the occurrence of neck pain in their study. Bertozzi L. et al. [65] also did not find a correlation between the angle of neck flexion while using a mobile phone and the occurrence of neck pain.

The differences in the above studies may stem from factors such as cultural or age-related differences among the participants. The studies by Raihan H.A. and Rahman F. [52] and Rashid M.K. et al. [26] were conducted in a population of adolescents and young adults attending Asian schools, while the studies by Correia I.M.T. et al. [64] and Bertozzi L. et al. [65] were conducted in a population of adult Europeans. Conducting research in a population that accounts for cultural differences could provide an interesting addition to the existing gap regarding the risk factors for the occurrence of text neck syndrome.

The relationship between smartphone addiction, the severity of neck pain, and the occurrence of text neck syndrome is confirmed by the study conducted by Mustafaoglu R. et al. [66]. A similar correlation has been found by Fatima S. et al. [67] and Shamsi R.F. et al. [17]. Gupta N. et al. [68], in a study of 957 students, have demonstrated that 22.4% of students have been found to be addicted to using electronic devices. In a study conducted by Paek K.S. [58] among 286 students, 15% were addicted to smartphones, and a trend was observed between addiction and the occurrence of neck pain. On the other hand, the study conducted by Alshahrani A. et al. [69] has demonstrated that as many as 60.3% of students at Qassim University were addicted to using smartphones, and a correlation has also been observed between addiction and the occurrence of cervical spine pain.

It is worth noting that neck pain affects women three times more often than men [61]. In turn, the analysis carried out by Chen Y.L. et al. [70] has proven that neck pain significantly more often affects women than men. Studies show that neck pain related to the text neck syndrome occurs more frequently in women [61,70]. An inverse relationship has been noted in the study by Kokiwar P.R. et al. [34] conducted among 306 students. Their study has shown a higher incidence of neck pain among men.

## 3. Occurrence of Text Neck

Approximately 30–50% of the adult population experiences neck pain [71]. The occurrence of neck pain has a tendency to increase [72]. However, those complaints are increasingly observed in adolescents and young adults. In the young adult population, the annual occurrence of neck pain ranges from 42% to 67% [72]. Epidemiological data indicate that pain syndromes in the cervical spine are one of the most common causes of disability and reduced quality of life in individuals aged 20–24 years [72]. Factors such as improper, forced body posture adopted while using electronic devices may contribute to an increased risk of neck pain [73].

Ayhualem S. et al. [1], in their publication, highlight that the risk of cervical spine pain is increased among students who spend much time using smartphones. They report that in a study conducted in Canada, 68% of students complained of musculoskeletal pain in the cervical spine related to smartphone use [1]. In a study conducted by Hassnain S. et al. [27] among 118 students in Lahore, the prevalence of text neck syndrome was very high, with 93.2% of the participants exhibiting symptoms of the syndrome. A high prevalence of text neck syndrome has also been noted in the research conducted by Javed A. et al. [28] and Khalid R. et al. [25].

Kumari S. et al. [20] have demonstrated the occurrence of text neck syndrome in 64.3% of the participants. A similar result has been obtained by Alsiwed K.T. et al. [29]—the text neck syndrome was present in 68.1% of the participants and in a study conducted by Rashid M.K. et al. [26]—64.5%. A low prevalence of text neck syndrome was observed in the study conducted by Kamaraj P.N. et al. [30] on a group of 354 individuals aged 18–22, where only 16.7% of the participants showed signs of smartphone-related neck syndrome. In turn, in a study conducted by Parmar N. et al. [31] among 428 individuals aged 17–24, 29.2% of them had text neck syndrome. A similar result was obtained in the study by Sathya P. and Tamboli S.A. [32], where among 100 physiotherapy students from Mumbai, aged 18–25, the occurrence of text neck syndrome was found in 32% of the students.

Atakla H.G. et al. [33] conducted a study in a significantly larger group than in the aforementioned studies. The researchers conducted a study among 1835 students at the University of Abomey Calavi in Benin. It has been found that 850 participants in the study exhibited symptoms of text neck syndrome. As shown by the cited studies, the prevalence of text neck syndrome in the adult population ranges from 16.7% to 93.2% [20,25,26,27,28,29,30,31,32,33].

## 4. Text Neck in Children

Due to the widespread availability of electronic devices, it is increasingly common for children to own mobile phones, giving them easier access to use those devices more frequently. According to Warda D.G. et al. [12], children aged 8–12 spend 41% of their time using electronic devices on smartphones, while adolescents aged 13–18 spend 46% of this time on smartphones.

Excessive smartphone use can be particularly harmful to children. While the young body is still developing, it receives inappropriate stimuli. Children may experience speech development issues, psychosocial disorders, vision problems, sensory integration disorders, or musculoskeletal disorders [11,12]. The last group includes, among others, text neck syndrome. The ratio of a child’s head size to body size is greater than in adults, which means the load placed on the cervical spine is higher in children than in adults, increasing the risk of developing text neck syndrome in that age group [12].

The issue of text neck syndrome in children and adolescents is addressed by Warda D.G. et al. [12], David D. et al. [11], and Jyothsna G. [74]. Children and adolescents spend an average of 5–7 h using smartphones, which puts them at an increased risk of experiencing issues related to text neck syndrome in the future [11]. It is estimated that the prevalence of neck pain in groups aged 7–11 years who are spending around 5–8 h daily using electronic devices is around 70% [23].

Neck pain is the eighth leading cause of disability among individuals aged 15 to 19 [13]. Musculoskeletal pain in the pediatric population shows similar patterns: specific symptoms (such as headache and musculoskeletal pain) are more prevalent in females than in males, and these rates tend to be higher in adolescents compared to children [11].

The above statements are confirmed by the research conducted by Fares J. et al. [75]. A study conducted by Fares J. et al. [75] in Beirut, Lebanon, involving patients under 18 years of age presenting with nonspecific neck pain. The average age of the participants was 14 years. In total, 207 children and adolescents were identified, with 180 diagnosed with musculoskeletal neck pain and spasms. The majority of participants (57%) were female, and more adolescents (60%) were affected compared to children (40%). All 180 participants reported flawed neck and back flexion while studying or using electronic devices such as smartphones and tablets. The study found a significant association between poor posture during technology use and musculoskeletal neck pain in adolescents [75].

Song M.K. at al. [76] conducted a study among 308 school-age children. The researchers have found that children with poor posture and those dependent on smartphones more often experienced neck and shoulder pain.

There are fewer reports of text neck syndrome among children than among adults. However, there have been publications describing case studies of children with text neck syndrome [11,43,77]. David D. et al. [11] described the occurrence of text neck based on a case study of a 16-year-old girl. Chu E. et al. [43] also described the prevalence of text neck syndrome in a case study of a 6-year-old boy. The boy underwent 9 months of chiropractic treatment, resulting in improved cervical spine mobility, reduction of neurological symptoms, and decreased pain. The researcher emphasizes the importance of early therapeutic intervention and prevention.

Aziz A.N. et al. [78] conducted a cross-sectional study aimed at assessing the prevalence of text neck syndrome and its associated factors in children and adolescents attending primary health centers (PHCs) in Erbil, Iraqi Kurdistan, in 2022. The study included children aged 5–15 years attending primary and secondary schools in Erbil. A total of 352 participants were recruited. The study found a high prevalence of text neck syndrome, with 69.0% of children reporting neck pain. Adolescents who used smartphones for more than 3 h daily (83.56%), those studying at the secondary school level, and those with psychological, social, and physical dysfunctions were more likely to develop text neck syndrome. Additionally, children who slept less, played fewer sports, used computers more, watched more TV, and played video games for more than 3 h were also at higher risk for text neck. The study concluded that the high prevalence of text neck syndrome is associated with increased smartphone use, screen time, and psychological and physical dysfunctions. The findings suggest the need for interventions to reduce screen time and promote better physical health practices in this population [78].

The study conducted by Rathi P. et al. [13] aimed to assess adolescents’ awareness, perceptions, and knowledge regarding the preventive measures, health hazards, and causes of text neck syndrome. The study included 302 adolescents from Pune city. The results revealed that 67.2% had heard of text neck syndrome, 47.7% were aware of its causes, and only 20.5% knew about holding smartphones at eye level. The study concluded that while awareness of text neck syndrome causes and health risks is good, knowledge of preventive measures remains limited, highlighting the need for enhanced educational efforts focused on prevention [13].

The authors emphasize that text neck syndrome is a serious problem that, if left untreated, may lead to significant health consequences for both children and adults. They highlight that prevention is the key, especially for children and adolescents [11,12,74].

Research on pain related to text neck syndrome in children is limited, with most existing studies focusing on young adults and college students. Therefore, additional research involving children and adolescents is necessary, particularly cross-cultural studies with large sample sizes.

## 5. Preventive Measures

Due to the increasing prevalence of text neck syndrome in the global population, the shifting age threshold, and the appearance of the condition in younger individuals, it is extremely important to raise awareness and knowledge about the consequences of improper excessive use of electronic devices.

The study conducted by Medani K.T. et al. [53] within a group of 229 students at Majmaah University has indicated that among the study participants, 71.2% have a low level of knowledge about text neck syndrome. On the other hand, the study conducted by Samani P.P. et al. [7] in a group of 311 people indicated that 35% of them had heard of text neck syndrome, but only 8% had knowledge about it. Accordingly, the study carried out by Rathi P. et al. [13] in a group of 302 teenagers has indicated that about 33% of the respondents have never heard of that term. The study conducted by Dolah J. et al. [79] has demonstrated that 39% of students are familiar with the term ‘text neck’, and the study conducted by Kumar S. et al. [22] among 200 respondents aged 18–25 has proven 50.3% of them to have no knowledge of text neck.

From a young age, children should be educated in schools about what text neck syndrome is, what the risk factors are, the health consequences of excessive and improper use of mobile devices, and what preventive and treatment methods are available [12]. A good solution could be the implementation of educational programs in schools or the use of smartphone applications that would inform users when they exceed the recommended screen time or adopt an incorrect head and neck posture during use [12,53].

Education is crucial both in prevention and in the therapeutic process. If a disorder has already occurred, the patient should be taught how to correct the posture, properly perform exercises, how to carry out self-therapy, modify movement habits, and adopt the correct posture when using electronic devices [5]. In addition, it is important to change harmful habits, adjust the workspace and study environment to individual needs, take regular breaks, and develop the awareness that the patient is responsible for the outcomes of their therapy and must follow recommendations if they wish to achieve results. Success requires perseverance, motivation, and commitment to the therapy.

The importance of education in the therapeutic process is emphasized by Farooq M. et al. [5]. Fouda K.Z. and Abdelsalam M.S. [80] also highlight that the combination of exercises to strengthen the deep flexor muscles of the neck with proper education and learning how to maintain a correct posture while using mobile devices significantly influences the achievement of positive treatment outcomes. A similar relationship between changing habits and therapy has been observed in the study by Shah J. and Soni K [54].

Regular physical activity is effective in preventing the occurrence of text neck syndrome [8,78]. Spinal stabilization exercises and exercises for maintaining a proper posture, when performed regularly, constitute an effective preventive measure [8,48].

Additionally, the position adopted while using a smartphone should be modified. Specifically, attention should be paid to holding the smartphone at eye level, thus preventing excessive neck flexion [12,40]. The study conducted by Namwongsa S. et al. [81] has evidenced that maintaining the neck at an angle of 0 to 15 degrees while using a smartphone significantly reduces neck muscle activity and the risk of developing neck pain.

In addition, activities that require frequent repetition of the same motion, such as texting or scrolling for extended periods, should be avoided [12,82]. It is also recommended to use both hands and index fingers when typing text messages, take frequent breaks every 20–30 min, frequently change positions, avoid using items that are too large and too heavy to hold in one hand for extended periods, and if necessary, use those items with arm support [12,40,82,83].

The study conducted by Vahedi Z. et al. [84] investigated the impact of smartphone use on musculoskeletal stress, specifically focusing on head forward flexion, lateral bending, and viewing distance during sitting/standing postures and one-handed/two-handed grips. The study monitored neck kinematics and evaluated performance in tasks such as typing, video watching, and reading. Results showed a significant increase in neck and upper limb pain after smartphone use. Sitting and standing postures were associated with greater head forward flexion and viewing distance during two-handed typing. One-handed tasks resulted in higher left lateral bending in standing posture. Overall, performance was better with two-handed grips [84]. These findings provide insights for recommending healthier smartphone usage practices.

It is also important to pay attention to reducing the amount of time spent using mobile phones. The Australian Department of Health guidelines from 2012 recommend that children under the age of 2 should not be exposed to mobile phones [12]. Children aged 2–5 years should not spend more than one hour per day using a mobile phone, while children and adolescents aged 5–17 years should limit their usage to 2 h per day during leisure time [12]. It is also worth mentioning that taking breaks and stretching during that time is an important preventive factor in the occurrence of unpleasant discomfort [48,74].

As reported by Elvan A. et al. [57], using a mobile phone for more than 3 h a day is a significant risk factor for the development of text neck syndrome. Shahzad Y. et al. [46] and Khan S. et al. [2] have demonstrated that neck pain occurs more frequently in individuals who spend more than 4 h a day using a mobile phone. De Vitta A. et al. [61], in a study conducted with a group of 1143 students, have also observed a correlation between the duration of smartphone use and the frequency of neck pain.

General recommendations suggest limiting non-essential screen time to 2–3 h per day for adults and less than 2 h for children and adolescents to reduce the risk of musculoskeletal issues. These recommendations are supported by the study by Aziz et al. [78]. Aziz et al. [78] conducted a cross-sectional study aimed at assessing the prevalence of text neck syndrome and its associated factors in children and adolescents in 2022. The study included children aged 5–15 years in Erbil. A total of 352 participants were recruited. Adolescents who used smartphones for more than 3 h daily were more likely to develop text neck syndrome. Additionally, children who slept less, played fewer sports, used computers more, watched more TV, and played video games for more than 3 h were also at higher risk for text neck. The findings suggest the need for interventions to reduce screen time and promote better physical health practices in this population [78].

The study conducted by Cevik S. et al. [85] aimed to explore the relationship between spinal degeneration parameters and increased smartphone usage time. Conducted as a cross-sectional study, it involved young adults aged 20–35 years. MRI was used to measure cervical disc degeneration, disc placement, Modic changes, and sagittal balance in 107 patients. Additionally, participants provided information about their daily smartphone usage through a questionnaire completed at the time of admission. Results suggested that smartphone use exceeding three hours daily had a greater impact on spinal degeneration parameters, including degenerative disc severity, disc placement, and Modic changes, compared to use for less than three hours [85].

The studies referenced in this chapter highlight the importance and relevance of the preventive guidelines for text neck syndrome. However, there is still a need for longitudinal cohort studies to assess the effectiveness of these preventive measures and validate their impact on reducing the risk of the condition.

## 6. Physical Therapy in Text Neck Syndrome—Literature Review

### 6.1. Literature Search

A scoping review was conducted on the treatment of text neck syndrome based on electronic databases: PubMed, ResearchGate, Physiotherapy Evidence Database (PEDro), and the Cochrane Library. The databases were searched up to 1 December 2024. The search phrases used were: “text neck syndrome”, “text neck”, “tech neck”, “tech neck syndrome”, “rehabilitation”, “physiotherapy”, and with the Boolean operator “and”. The search strategy for the databases is presented in Table 2. Data were independently charted by one reviewer.

The inclusion and exclusion criteria for the review are presented in Table 3. The review included clinical trials and experimental studies on rehabilitation methods used in patients with text neck syndrome, published between 2018 and 2024 and written in English and in full text. Papers were excluded if they did not align with the conceptual framework of the study.

### 6.2. Included Studies

**Figure 2:**
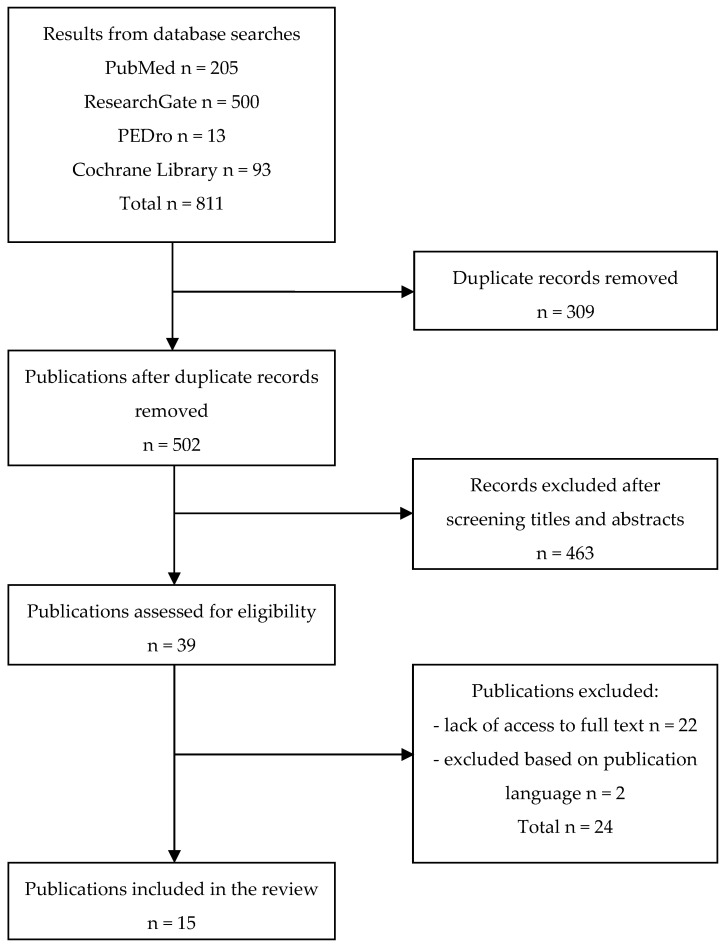
Flow diagram demonstrating the scoping review process.

As a result of searching the literature, 811 records were found. After removing duplicates, 502 publications were eligible for screening. Following title and abstract screening, 39 articles were eligible for full-text assessment. After meeting the inclusion criteria, 15 articles qualified for the review. Figure 2 presents a flow diagram demonstrating the scoping review process.

The latest literature highlights the use of, among others, postural correction exercises, stabilization exercises, strengthening and stretching exercises, Pilates, PNF (Proprioceptive Neuromuscular Facilitation), Kinesiology Taping, Bowen therapy, and manual therapy in the rehabilitation of text neck syndrome [5,19,23,38,39,42,50,86,87,88,89,90,91,92]. The summary of the latest research on the use of rehabilitation methods in the treatment of text neck syndrome is presented in Table 4.

All studies were published within the last seven years. Most studies focused on adult populations aged 18–35, with three studies including individuals aged 18–44 [19,42,50] and one study involving adolescents [92]. The majority of studies were conducted in Asia, including countries such as India, Thailand, Pakistan, Iran, and Turkey in Eurasia [5,19,35,38,39,42,50,86,87,88,89,90,91,92].

In most studies, no funding was received (80%) [19,35,38,39,86,87,88,89,90,91,92], with only three studies receiving funding [5,42,50].

The physiotherapy methods identified as useful and effective in reducing pain and correcting posture in individuals with text neck syndrome include PNF (Proprioceptive Neuromuscular Facilitation) [19,86], corrective exercises, strengthening the deep neck flexor muscles [87], dynamic correction exercises [49], Pilates [23,90], Gong’s mobilization [88], ELDOA [5], and kinesiology taping (KT) [91,92].

Regarding the improvement of cervical range of motion (CROM), the most effective methods were PNF [19,86], Gong’s mobilization [88], and Pilates [23,90].

### 6.3. Summary of Current Evidence

Due to the fact that text neck syndrome primarily affects young adults, i.e., the working population, the symptoms associated with that syndrome may significantly impact the deterioration of their functioning and quality of life [1,93,94,95,96]. Therefore, early implementation of treatment is crucial to prevent the progression of symptoms, avoid degenerative changes in the cervical spine, and further impairment of function. In order to minimize the negative effects and reverse the symptoms of that syndrome as much as possible, various physiotherapeutic methods are used.

Depending on the severity of symptoms, different forms of therapy can be applied. For acute symptoms of text neck syndrome, rest, hot or cold compresses, massage, stretching, exercises, postural correction, and lifestyle changes are recommended. For chronic symptoms of text neck syndrome, physical therapy treatments, painkillers, injections, and acupuncture are used [5].

The duration of physical therapy for text neck syndrome varies depending on the severity of the condition. On average, for mild cases, physical therapy may last for 2–4 weeks with exercises aimed at improving posture, strengthening muscles, and increasing flexibility. In more severe cases, physical therapy could be required for 6–8 weeks or longer, with ongoing exercises and possibly additional treatments like manual therapy. Early intervention often leads to quicker recovery, while chronic cases may require longer treatment [5,19,23,38,39,42,50,86,87,88,89,90,91,92].

The main goal of physiotherapy treatment is to eliminate the problem and, if that is not possible, to reduce the perceived discomfort and prevent the disorder from progressing [5,90,97]. That goal may be achieved through the normalization of muscle tension, improvement of mobility in the cervical spine joints, enhancement of tissue trophism, and biomechanics [5]. An individually tailored therapeutic program may bring numerous benefits.

The scoping review was aimed to determine the physiotherapy methods currently used in the treatment of individuals with text neck, assess their impact on symptom reduction, and identify existing knowledge gaps and limitations in the current literature on the rehabilitation of text neck syndrome.

This review analyzes studies from the last 7 years, written in English, where the full text was available and clearly stated that the participants had text neck syndrome. The reviewed studies examined the effectiveness of the following rehabilitation methods: postural correction exercises, stabilization exercises, strengthening and stretching exercises, Pilates, PNF (Proprioceptive Neuromuscular Facilitation), Kinesiology taping, Bowen therapy, and manual therapy in the rehabilitation of text neck syndrome [5,19,23,38,39,42,50,86,87,88,89,90,91,92].

Before any therapeutic intervention is applied, it is essential to conduct diagnostics to identify abnormalities and functional disorders, which will enable the creation of an appropriate rehabilitation plan [38]. Detailed diagnostics should facilitate the identification of ‘red flags’ that indicate serious dysfunctions, such as the presence of a tumor or atlanto-axial subluxations. After a thorough assessment, the physiotherapist sets rehabilitation goals and establishes a treatment plan that is modified according to the patient’s progress and condition changes [74]. The selection of therapy forms, methods, and techniques depends on the patient’s current health status, reported symptoms, and coexisting conditions.

#### 6.3.1. Kinesiotherapy

In the therapy of text neck syndrome, exercises should be carefully selected to normalize muscle tension. The forward shift of the head’s center of gravity in text neck syndrome places increased strain on the cervical spine joints and muscles, such as the cervical extensor muscle and the upper part of the trapezius muscle. At the same time, deep neck flexor muscles, such as the longus capitis, longus colli, and rectus capitis anterior, lose their strength and endurance. Such a muscle imbalance may contribute to pain and dysfunction, which is why targeted exercises aim to strengthen the weakened muscles while relieving the overburdened ones [18]. The rhomboid muscles, as well as the middle and lower portions of the trapezius muscle, also weaken [18]. The pectoralis minor muscles and muscles of the rotator cuff are subjected to tension [18]. Due to that, it is recommended to incorporate exercises into the therapy that strengthen weakened muscles, such as deep neck flexors and stretching exercises for the shortened muscles [80].

Based on the analysis of available studies, there is noticeable effectiveness of stabilizing, corrective, and strengthening exercises for deep neck flexors in the treatment of text neck syndrome [19,38,42,50,86,87,89,90]. It is important to emphasize that adopting the appropriate position, duration, intensity, frequency, and difficulty level, tailored to the individual needs of the patient as well as health conditions is crucial in these exercises [38,42].

Sarraf F. et al. [87] demonstrated the effectiveness of corrective exercises in reducing pain and the NDI (Neck Disability Index) score in their study. The researchers have been able to achieve that effect because the corrective exercises contributed to strengthening the deep neck flexor muscles and stretching the tense muscles in both the neck and shoulder girdle areas [87]. Stretching and strengthening exercises are effective in maintaining the correct position of the cervical spine and improving stability in individuals with text neck syndrome [19,38,50,87]. The research conducted by Kang D.Y. [98] and Agrawal Y.K. and Hande D. [99] confirms that exercises targeting the deep neck flexors are highly effective in restoring the muscle tension balance and, consequently, alleviating pain in that area.

Wissem D.W. and Saadc H.B. [49] emphasize the superiority of dynamic correction over static postural correction exercises. Traditional static postural correction exercises focus on restoring and maintaining the cervical spine in a neutral position and adhering to ergonomic principles [49]. On the other hand, dynamic correction is based on the joint-by-joint approach, which focuses on restoring mobility and stability throughout the entire kinetic chain, including the thoracic spine and shoulder girdle, rather than just focusing on the cervical spine [49]. That approach provides a long-term effect rather than temporary relief, as is the case with traditional static postural correction exercises [49]. According to the joint-by-joint approach, the therapeutic process for text neck should include mobilization of the thoracic spine, stabilization exercises, scapular control exercises, and exercises incorporating rotational movements in the thoracic spine [49].

In order to improve the patient’s functionality, the proprioceptive neuromuscular facilitation (PNF) method is also used, which utilizes developed movement patterns [19]. PNF is based on neurophysiological principles, using tri-planar and diagonal movement [19,86]. In a study conducted by Kaya M. et al. [19] on a group of 38 individuals with text neck, comparing the use of exercises improving CROM, strengthening, and postural correction with the application of PNF techniques—contract–relax and replication—a statistically significant better improvement in the range of motion of the cervical spine was observed in the group using PNF. It may be due to the fact that PNF techniques improve the range of motion by activating both agonist and antagonist muscles, which helps lengthen muscles and tendons. PNF, particularly the contract–relax and hold–relax methods, is often more effective than static stretching due to mechanisms like reciprocal and autogenic inhibition. During PNF, when a muscle contracts against resistance, tension builds up, activating Golgi tendon organs, which then reduce muscle tension by inhibiting excessive contraction. This process helps improve flexibility and range of motion, as supported by this study [19].

Similar results were obtained in a study conducted by Rajopadhye S. and Honkalas P. [86], evaluating the effectiveness of the Dynamic Reversal technique for improving the range of motion of the cervical spine and the Rhythmic Stabilization technique for reducing neck pain in individuals with text neck syndrome. After eight therapeutic sessions conducted over 2 weeks, the researchers observed statistically significant improvements in the measured parameters [86].

The positive effects on pain reduction in the PNF group may be due to the activation of impaired proprioception centers through various PNF techniques. These techniques stimulate proprioceptive receptors in muscles and tendons, improving nerve control, muscle tone, and circulation. Additionally, the decrease in pain intensity could be linked to the local afferent input generated by PNF interventions, which help modulate pain perception [19,86].

It is important to note that the key to success is to perform exercises correctly and safely. The therapist should provide guidance on how to exercise. The duration, type, intensity, frequency, and level of difficulty of the exercises should be tailored individually to the patient, health status, functional abilities, and preferences. In order to achieve benefits such as an improved range of motion and tissue nourishment, increased functional capabilities, alleviation of pain, and improved posture, it is necessary to work with the entire body of the patient, eliminate coexisting dysfunctions rather than just working locally [83,97].

Shah J. and Soni K. [90] recommend incorporating Pilates into the therapy for text neck syndrome. The Pilates method positively influences psychophysical functioning, strengthens muscles, improves core stabilization, and enhances mobility, balance, flexibility, and endurance [23,90]. The effectiveness of using Pilates in therapy for text neck syndrome was evaluated by Bhanu Sri P.L. et al. [23]. The researchers evaluated the impact of the Gong mobilization technique and Pilates on pain intensity, range of motion, and function. It has been shown that after 6 weeks of therapy, a greater improvement in the studied parameters was observed in the Pilates group [23]. Pilates helps activate the deep neck flexor muscles—it strengthens and improves the endurance of the deep muscles, thereby relieving and reducing the activity and fatigue of the superficial muscles, which in turn leads to pain reduction and improvement of overall functioning [23,50,90].

Pilates promotes a neutral cervical spine position with slight flexion at the cranio-cervical junction, activating the deep neck flexor muscles. By combining breathing techniques with specific stretches, Pilates helps manage neck pain. Neck pain is often linked to weakness in the deep cervical flexors, and Pilates aids in re-educating the stabilizing muscles of the spine and shoulder girdle. This not only alleviates pain but also improves function by strengthening and increasing endurance in the cervical muscles [23,90].

In Pilates, it is important to combine exercises with proper breath control [90]. Shikha B. et al. [35] emphasize the importance of breath work in the treatment of text neck syndrome. The researchers compared the effectiveness of balloon-blowing exercises with modified neck exercises and exercises using a Swedish ball on lung capacity. After 4 weeks of therapy, improvement in the measured parameters (FEV, FVC) was observed in both groups, with a greater improvement in the group performing balloon-blowing exercises [35].

Bhende R. et al. [42] investigated whether integrated postural training, focusing on the involvement of three systems—visual, vestibular, and somatosensory—that coordinate postural control of the neck, would be effective in treating text neck syndrome. The training included neck proprioception exercises, deep neck muscle strengthening exercises, and strengthening exercises; the McKenzie program focused on postural correction, as well as oculomotor exercises and eye–head coordination exercises [42]. The authors have demonstrated that a holistic approach, rather than merely addressing symptomatic relief, offers numerous benefits. A six-week integrated postural training program for individuals with text neck syndrome resulted in improved posture, strengthened weakened muscles, restored joint mobility, reduced pain, and enhanced overall functional capacity [42].

In turn, Varyani S. et al. [89] focused on applying a conventional exercise program that included isometric neck exercises combined with eye muscle exercises for individuals with text neck syndrome. In the study group of 36 participants, the researchers observed improvements in neck flexor muscle endurance, pain reduction, alleviation of asthenopic symptoms, and enhanced functionality after four weeks of therapy [89].

Many authors emphasize that combining physiotherapeutic methods yields better results than using individual treatments alone [5,19,39,42]. Nakhate S. et al. [48] recommend implementing a 1–4-week rehabilitation program that should begin with soft tissue mobilization, followed by joint mobilization and both active and passive stretching of tense muscles. The next recommended steps include strengthening weakened muscles, correcting posture, changing habits, performing home exercises, and reducing time spent using a phone [48].

#### 6.3.2. Manual Therapy

Manual therapy is used to increase the range of motion, restore proper joint mechanics, normalize the tension of paraspinal tissues, and reduce pain during movement [5,100]. Manual therapy not only addresses the cervical spine but also works on the thoracic spine and the cervicothoracic junction [49]. It may include manipulations, mobilizations, or Muscle Energy Techniques (MET) [5].

The muscle energy technique is a form of manual therapy aimed at improving joint mobility by lengthening tense muscles and strengthening weakened muscles through the use of isotonic or isometric contractions [39]. The Bowen therapy, on the other hand, employs gentle rolling movements applied to soft tissues in a specific manner and at designated points [39].

The effectiveness of the MET and Bowen therapy in the treatment of text neck syndrome was assessed by Seemal P. et al. [39]. The researchers observed that the combination of the Muscle Energy Technique and Bowen therapy yielded better results than using the MET alone in the treatment. The combination of both therapies resulted in a statistically significant reduction in perceived pain as well as improvements in the function and mobility of the cervical spine [39]. Afzal H. et al. [88] also demonstrated the effectiveness of manual therapy—Gong’s mobilization in the reduction of neck pain, disability, and improvement of CROM and neck muscle strength in individuals with text neck syndrome.

The effectiveness of manual therapy has also been demonstrated in individual case reports of people with text neck syndrome [37,43]. One study focused on the use of manual therapy and instrument-assisted therapy in a 6-year-old boy with text neck syndrome [43]. The treatment applied resulted in a reduction of pain and neurological symptoms and an improvement in the Cervical Range of Motion (CROM) [43].

Farooq M. et al. [5] demonstrated the effectiveness of the ELDOA (Elongation Longitudinaux Avec Decoapton Osteo-Articulaire) method and post-facilitation stretching in a group of 40 participants with text neck syndrome, aged 18–35. The ELDOA group and the stretching group each consisted of 20 individuals. Each group received the assigned therapy three times a week for 6 weeks. Both groups showed a reduction in neck pain and NDI scores, but the ELDOA group showed a statistically significantly greater improvement than the post-facilitation stretching group.

Better results in the ELDOA group may be related to the fact that ELDOA focuses on maximizing spinal and fascial stretching by holding specific postures. This technique aims to strengthen and decompress the spine. Originating from various treatment methods, ELDOA has both local and overall effects. It helps decompress the zygapophyseal joints, enhances fluid absorption in the discs, improves circulation, and boosts muscle tone and range of motion. Additionally, it aids in correcting posture, improving breathing, and enhancing proprioception in the targeted segment [5].

#### 6.3.3. Kinesiology Taping

In the rehabilitation of text neck syndrome, the Kinesiology Taping (KT) method is used. It involves applying an elastic tape with a specific structure to the skin surface using a technique appropriately selected for the therapeutic goal. The application of the tape is performed after properly preparing the patient’s skin for the application. The pain-relieving effect is achieved by improving the microcirculation of blood and lymph, activating alpha and beta nerve fibers and their skin connections, and unloading the subcutaneous nociceptors [91,92]. In addition to its analgesic effect, Kinesiology Taping influences the normalization of muscle tone, improvement of posture, and stabilization of the achieved therapeutic effects—maintaining the corrected position [92]. For example, in individuals with head protrusion, excessive stretching of the extensors and neck pain may occur, so the use of KT will, in such a case, serve as a supportive function for the extensor muscles in maintaining the head in the correct position.

However, it should be noted that Kinesiology Taping is not used as a standalone method in the treatment of text neck syndrome. It serves as an attractive complement to other therapeutic methods; for example, it may be used to maintain the effects of manual therapy [91,92,101].

KT is a complementary form that supports proper treatment [91,92]. Kothare H. et al. [91] have noted that the appropriate application of KT may be effective in alleviating the symptoms of text neck syndrome as early as 3 days after application. The effectiveness of KT in reducing pain symptoms is supported by studies conducted by Areeudomwong P. et al. [92]. The authors of the study examined whether shoulder taping would affect the perceived discomfort in the neck area as well as the activity and endurance of the upper part of the trapezius muscle, neck extensors, and sternocleidomastoid muscles during 30 min of smartphone use. The results have demonstrated that KT reduced pain and discomfort but had no impact on muscle activity or fatigue [92]. Shoulder taping was found to reduce neck discomfort compared to no taping. While the exact mechanisms behind this effect are not fully understood, it is possible that the tension from the tape plays a role in alleviating discomfort. Participants who received shoulder taping reported only moderate skin tension, which may activate skin mechanoreceptors and reduce pain through the gate control theory, thus lessening their discomfort [92].

### 6.4. Limitations of the Included Studies

One of the primary limitations identified in the cited literature on physical therapy for text neck syndrome is the lack of blinding of both the researchers and the participants [5,19,35,38,42,50,86,87,88,89,90,91,92]. Additionally, most of the studies were conducted with a young adult population [5,35,38,86,87,88,89,90,91], which may not be representative of other age groups. The studies also utilized small sample sizes, limiting the generalizability of the results to a larger population. Furthermore, the studies were geographically restricted, which may reduce the applicability of the findings to other regions. The majority of studies were conducted in India [23,35,38,42,50,86,89,90,91]. Lastly, the studies were short-duration and lacked long-term follow-up, making it difficult to assess the sustained effects of the interventions.

### 6.5. Limitations of the Study

In a scoping review, the process of selecting, evaluating, and synthesizing the included studies may be less rigorous and potentially prone to bias, as it generally does not involve a formal quality assessment of the literature. Nevertheless, the strength of this type of review lies in its ability to provide a clear and accessible summary and critique of existing literature, providing valuable new insights. To enhance the methodological rigor of our review, we have clearly defined the eligibility criteria, search strategies, and databases used, as well as specified the number of records to be included, which helps to better outline the scope of the study.

This review has analyzed publications written in English for the last seven years regarding the use of physical therapy in text neck syndrome, which may lead to bias in study selection due to the lack of data on text neck syndrome described in languages other than English. Additionally, many articles are not publicly available. As a result, our findings can only be generalized to studies that are publicly available, which is a limitation of the study. Despite these limitations, this scoping review serves as a valuable resource for understanding the current state of research on the rehabilitation of text neck syndrome.

### 6.6. Future Research Objectives

This review highlights several key areas for future research on text neck syndrome. Most existing studies have focused on young adults and college students, leaving gaps in research regarding other age groups. Notably, there is a lack of studies on children and adolescents, an area that warrants further investigation. Research on pain related to text neck syndrome in children is limited, and establishing its prevalence along with clear diagnostic criteria using validated tools is a crucial step. Developing a standardized instrument for assessing text neck syndrome in the pediatric population would enable cross-cultural comparisons and facilitate the comparison of different sample sizes, accounting for cultural differences. As a result, cross-cultural studies involving large sample sizes are recommended to improve the generalizability and applicability across various populations.

Furthermore, long-term cohort studies are essential for evaluating the effectiveness of preventive measures and assessing the lasting impact of interventions. The need for longitudinal studies with long-term follow-up periods is critical to understanding the sustained effects of interventions over time.

Recommendations for future research on physical therapy in text neck syndrome include conducting longitudinal studies with extended follow-up periods to observe the long-term effects of interventions. Increasing sample sizes and conducting studies with diverse populations, including children, adolescents, and elderly individuals, is also essential. Expanding the geographical scope and incorporating cross-cultural studies to improve the generalizability of findings is crucial. Utilizing randomized controlled trials (RCTs) would be a good approach to strengthen the evidence base.

## 7. Conclusions

It is not possible to eliminate electronic devices from daily use, so it is important to maintain a proper posture while using them, reduce the time spent using smartphones, and ensure regular physical activity. When text neck syndrome does occur, it is crucial to start treatment promptly to prevent further functional decline and irreversible changes. The studies cited in this paper indicate the effectiveness of physiotherapy in treating text neck syndrome. All studies suggest that appropriate physiotherapeutic interventions can provide significant benefits, including pain reduction, posture correction, and improved range of motion in the cervical spine. The most effective therapeutic approach involves combining various rehabilitation methods, including postural correction exercises, stabilization exercises, strengthening and stretching exercises, Pilates, PNF (Proprioceptive Neuromuscular Facilitation), kinesiology taping, Bowen therapy, and manual therapy. The best treatment outcomes are achieved through a combination of strengthening and stretching exercises, manual therapy, and postural correction. In addition to physiotherapy, education is essential at every stage, both in prevention and treatment. However, further high-quality research is needed to strengthen the evidence and offer reliable recommendations for clinical practice. Additionally, there is limited research on physiotherapy for text neck in the pediatric population, presenting a potential area for future studies.

## Figures and Tables

**Table 1:** Symptoms and consequences of text neck syndrome.

Typical Symptoms	Additional Symptoms	Health Consequences
- Muscular tension imbalance - Neck pain - Neck stiffness - Neck soreness - Flattening of the cervical lordosis	- Radiating pain in the upper limbs - Upper back pain - Shoulder girdle pain - Headaches - Tingling in the hands - Numbness in the hands - Muscle weakness - Sleeplessness - Impaired range of motion in the cervical spine.	- Pressure on spinal nerves - Nerve and muscle damage - Cervical proprioception disturbances - Impaired range of motion in the thoracic spine - Degenerative changes - Herniated discs - Disruption of the spine’s physiological curves - Reduced lung capacity - Cervical-origin dizziness - Early-onset arthritis - Disorders in the temporomandibular joint.

Own elaboration based on [6,7,19,25,34,37,39,40,47,48,49,50].

**Table 2:** The search strategy for the databases.

PEDro	Cochrane Library	PubMed	ResearchGate
- text neck and physiotherapy - text neck and rehabilitation - tech neck and physiotherapy - tech neck and rehabilitation - text neck syndrome - tech neck - text neck syndrome and rehabilitation - text neck syndrome and physiotherapy	- text neck and physiotherapy - text neck and rehabilitation - tech neck and physiotherapy - tech neck and rehabilitation - text neck syndrome - tech neck	- text neck syndrome and physiotherapy - text neck and rehabilitation - tech neck syndrome and physiotherapy - text neck syndrome - tech neck syndrome	- text neck and physiotherapy - text neck and rehabilitation - tech neck and physiotherapy - text neck syndrome - tech neck syndrome

**Table 3:** Inclusion and exclusion criteria for review.

Inclusion Criteria	Exclusion Criteria
- year of publication: 2018–2024 - language: publications written in English - type of study: clinical trial, experimental study - topic—rehabilitation in text neck syndrome - full text available	- year of publication: below 2018 - language: publications written in languages other than English - systematic review, case study - topics not of main interest - duplication of other publications already included in the review - full text not available

**Table 4:** The summary of the latest research on the use of rehabilitation methods in the treatment of text neck syndrome.

Primary Author (Year of Publication)	Number of Study Participants	Intervention	Intervention Duration	Studied Parameters	Results
Kaya M. (2024) [19]	n = 38	G1—exercises to improve CROM, strengthen, and correct posture. G2—exercises to improve CROM, strengthen, and correct posture, as well as PNF (contract–relax technique, replication technique)	Exercises in both groups: 10 repetitions, once a day, 3 days a week; PNF: 3 days a week; Duration: 6 weeks	VAS, CROM, NFEMET, NYPRC, NDI	Improvement of the studied parameters in both groups, but greater improvement in G2. CROM improvement was observed in G2.
Bhende R. (2024) [42]	n = 80	G1—conventional exercise program G2—integrated postural training	3 times a week; 6 weeks	VAS, NDI, CROM, TTW, CPJE Testing	Improvement of parameters in both groups, but CPJE testing was better in G2 than in G1.
Nathani H.R. (2024) [38]	n = 54	Individually tailored rehabilitation protocol	3 weeks	VAS, NDI, SAS, CHDQ, ROM	Improvement in VAS, NDI, and CHDQ after the treatments.
Rajopadhye S. (2023) [86]	n = 63	PNF (Dynamic Reversal Technique, Rhythmic Stabilization Technique)	4 times a week, 2 weeks	VAS, CROM (cervical spine extension)	Improvement in CROM and pain reduction.
Bharal S. (2023) [50]	n = 75	G1—isometric neck exercises G2—neck stabilization training G3—Contrology training	4 weeks	CVA, NPRS, NDI	Better results in G2 and G3 than in G1. CVA increased, and NPRS and NDI decreased. In G2, better results than in G3.
Bhanu Sri P.L. (2023) [23]	n = 60	G1—Pilates, G2—Gong’s Mobilization	5 sessions a week; 6 weeks	VAS, NDI, CROM	Improvement of parameters in both groups, Better results in G1.
Sarraf F. (2023) [87]	n = 60	G1—corrective exercises G2—no therapy	1 daily, 5 times a week; 8 weeks	NDI, VAS, photogrammetry	Improvement in posture and VAS, NDI parameters in G1.
Afzal H. (2023) [88]	n = 24	G1—Kendall’s intervention G2—Gong’s mobilization	3 times a week; 6 weeks	NDI, NPRS, CROM, CVA, RSA, neck muscle strength	Greater improvement in parameters in G2.
Farooq M. (2023) [5]	n = 40	G1—10 min hot pack session, ELDOA, ergonomic recommendation, G2—10-minute hot pack session, post-facilitation stretching, ergonomic recommendation.	3 sessions weekly, 45 min each, 6 weeks	NPRS, NDI, SAS	Greater improvement in parameters in G1.
Seemal P. (2022) [39]	n = 22	G1—hot packs and METs G2—hot packs, METs, and Bowen therapy	3 sessions weekly; 6 weeks	CROM, CVA, NPRS, NDI, RSA	Improvement of the studied parameters in both groups, but better results in G2.
Shikha B. (2022) [35]	n = 30	G1—balloon blowing activity, G2—modified cervical exercise along with Swiss ball exercise.	4 weeks	FEV, FVC (spirometry)	Improvement in FEV and FVC in both groups, but better results in G1.
Varyani S. (2022) [89]	n = 36	Traditional exercises (neck strengthening exercises, upper body stretching, side bends with chin tuck, chin tucks) and eye muscle exercises.	25–30 min daily, 5 times a week; 4 weeks	NDI, OSDI, NPRS	Reduction of neck pain, improvement in NDI and OSDI.
Shah J. (2019) [90]	n = 30	G1—conventional exercise program, ergonomic advice G2—the same as above, plus Pilates	6 weeks	NPRS, NDI, neck muscle strength and endurance	Improvement of the studied parameters in both groups.
Kothare H. (2019) [91]	n = 50	KT application on UT	3 days	NPRS, NDI	Improvement of the studied parameters.
Areeudomwong P. (2018) [92]	n = 25	Two conditions: - shoulder taping, - no taping	30 min	NPRS, muscle activity, and fatigue during smartphone writing were assessed using electromyography	Reduction of pain and discomfort with KT application. No effect of KT on muscle activity and fatigue.

Abbreviations: G1—group 1; G2—group 2; CROM—Cervical Range of Motion; PNF—Proprioceptive Neuromuscular Facilitation; VAS—Visual Analogue Scale; NFMET—Neck flexor-extensor muscle endurance test; NYPRC—New York Posture Rating Chart; NDI—Neck Disability Index; TTW—tragus to wall test; CPJE Testing—Vernier Caliper measurements for rounded shoulders and cervical joint position error testing; SAS—Smartphone Addiction Scale; CHDQ—Cornell Hand Discomfort Questionnaire; ROM—range of motion; G3—group 3; CVA—Craniovertebral angle; NPRS—Numerical Pain Rating Scale; RSA—Rounded Shoulder Angle; ELDOA—Elongation Longitudinaux Avec Decoapton Osteo-Articulaire; METs—Muscle energy technique; FEV—Forced Expiratory Volume; FVC—Forced Vital Capacity; OSDI—Ocular Surface Disease Index; KT—Kinesiology Taping; UT—upper trapezius.
